# Phytochemical Profiling and Larval Control of* Erythrina variegata* Methanol Fraction against Malarial and Filarial Vector

**DOI:** 10.1155/2019/2641959

**Published:** 2019-04-16

**Authors:** Mathalaimuthu Baranitharan, Barbara Sawicka, Jayapal Gokulakrishnan

**Affiliations:** ^1^Department of Zoology, Annamalai University, Annamalainagar 608 002, Tamilnadu, India; ^2^Department of Plant Production Technology and Commodities Science, University of Life Sciences in Lublin, Akademicka 15 Str., 20-950 Lublin, Poland; ^3^P.G & Research Department of Zoology, Poompuhar College, Melaiyur 609 107, Tamil Nadu, India

## Abstract

*Erythrina variegata* (*E. variegata*) bioactive chemical has been the potential to be utilized as a good, eco-friendly approach for the control of mosquito population. In the present investigation, methanol extract using insecticidal compounds isolated against mosquito larvae kill assay was carried out. Secondary metabolism was characterized by thin layer chromatography, column chromatography, Fourier transform-infrared spectroscopy, gas chromatography-mass spectral, and identification of compound. Mosquito immature third instar larval,* Anopheles stephensi,* and* Culex quinquefasciatus* have been exposed to different concentrations of 50-250 *µ*g/ml. Totally, larvae were death rate 98.2% (significant value 0.001^b^) from methanol extract and it is significant toxicity against larvae of* An. stephensi* and* Cx. quinquefasciatus* with LC_50_/LC_99_ values were 157.69/339.55 *µ*g/ml and 137.67/297.33 *µ*g/ml, respectively. FT-IR analysis in the functional groups such as alcohol, amines, amides, alkenes, 1^⁎^ amines, aromatic amines, aliphatic amines, 1^⁎^,2^⁎^ amines, and alkyl halides searched the identity of secondary metabolites, which may act as 12-Octadecenoic acid, methyl ester compound and clearly indicates being phytochemical. Chemical constituents of twenty-five compounds were identified in the methanol extract. The major components were 12-Octadecenoic acid and methyl ester (37.31%). Compound molecules consist of carbon 19 atoms (gray), hydrogen 36 atoms (greenish blue), and oxygen 2 atoms (red), indicated by the different colors. The results were obtained suggesting that, in addition to their pharmaceutical and medicine sources, 12-Octadecenoic acid, methyl ester compound can also serve as a natural mosquito control.

## 1. Introduction

Mosquitoes are very well recognized as vectors of protozoan, viruses, and other pathogenic organisms and it is well-known also that under the influence of environmental conditions a vector species may notice changes in the seasonal distribution in the same area of dominance. The increase in density of a vector species is very much dependent on climatologically factors favorable for its breeding and adult survival. The effects of land use change by humans have long been recognized as a factor in the exacerbation of mosquitoes and mosquito-borne diseases [1]. Biological invasions challenge our ability to understand the biotic and abiotic process that governs their distribution and abundance [2]. Different mosquito species exhibit particular type of rhythmic pattern of behavior during their life cycle. Majority of the mosquito's rest during day time and their activities start little before the dusk and end little after the dawn. Majority of the* Anopheles* and* Culex* species are night time biters. Feeding host preference of mosquitoes varies from human to other mammals and birds. Mosquitoes being vector for many tropical and subtropical diseases are the most important single group of insects well-known for their public health importance. Despite progress in vaccine development, no effective and acceptable multivalent vaccines are currently available against vector borne diseases [3]. The approach to combat these diseases largely relies on interruption of the aquatic stages or by killing the adult mosquitoes using chemical insecticides. The drastic effects of chemical insecticide based intervention measures for the control of disease vectors have received wide public apprehension and have caused many problems like insecticide resistance, resurgence of pest species, and environmental organisms. Plants are rich sources of bioactive compounds that can be used to develop environmentally safe vector and pest managing agents. Botanical phytochemicals with mosquitocidal potential are now recognized as potent alternative insecticides to replace synthetic insecticides in mosquito control processes due to their excellent larvicides, ovicides, and adulticides properties [4].

In recent years, important efforts have been managed to propose plant-borne compounds as important alternatives to synthetic mosquitocides, due to their effectiveness, reduced toxicity towards vertebrates, and high biodegradability [5]. In* Erythrina variegata* (*E. variegata*) (Vernacular name: Kalyana murungai in Tamil), siddha medicine is used especially for menstrual disorders and fissures at penis tip [6]. The bark and root were noticed the presence of glycosides, volatile oils, carbohydrates, and tannins. Furthermore, seeds yield a saponaceous glucoside, a fatty oil, and alkaloid. Leaves and bark yield a poisonous alkaloid. The leaves, bark, and root are used in India for the treatment of various diseases and* E. variegata* shows antiosteoporotic [7], cytotoxic [8], anthelmintic [9], antiulcer [10], diuretic [11], analgesic [12], cardiovascular effect, respiratory effect [13], and antioxidant activity [14]. In present research, we assessed the mosquitocidal action of* E. variegata* methanol extract and 12-Octadecenoic acid, methyl ester compound on* An. stephensi* and* Cx. quinquefasciatus.*

## 2. Materials and Methods

### 2.1. Sample Collection and Preparation

**Figure 1:**
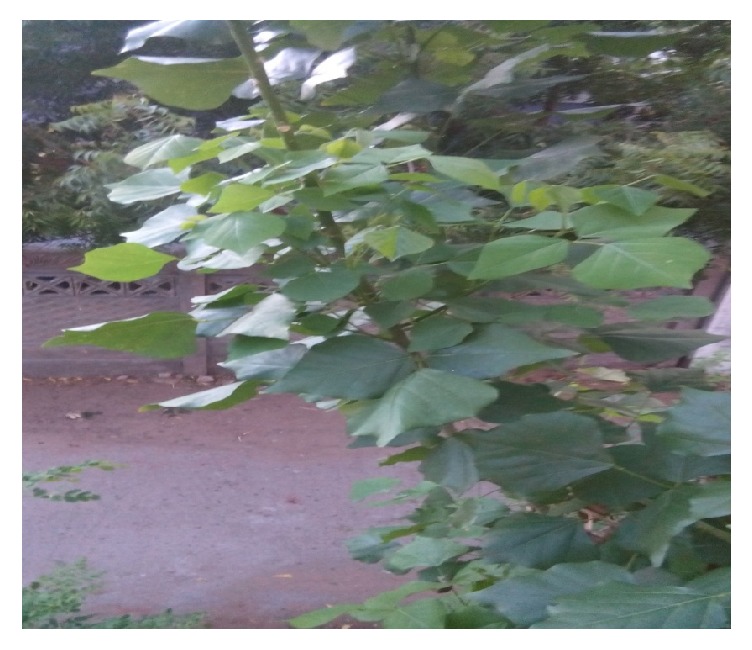
Medicinal plants,* Erythrina variegate*.

New, developed leaves of* E. variegata* (Figure 1) are collected from the Velankanni (10°40′49.09′′N latitude and 79°50′58.91′′E longitude), Nagapattinam District, Tamilnadu, in India. Plant is legitimately validated in the Department of Botany, Annamalai University. Plant leaves were air-dried in the dark place and each sample was ground to a fine powder. The dried leaves (100g) were powdery automatically exploitation business electrical stainless-steel liquidizer and extracted consecutive with methanol, ethanol, chloroform, and acetone exploitation Soxhlet equipment. The extract was focused underneath reduced pressure 22–26 mmHg at 45°C by ‘Rotavapour' and therefore the residue obtained was held on at 4°C. The condensed crude leaves extract was held on in refrigerator till needed for investigation for larvicidal, ovicidal, and repellent activities.

### 2.2. Culture of Mosquitoes

The mosquito vectors,* An. stephensi* and* Cx. quinquefasciatus* larvae, were gathered from restorative field and stagnant water zones of Chidambaram, Tamilnadu, in India. It was kept up at 27±20°C, 75-85 relative mugginess. The hatchlings were bolstered with dog biscuits and yeast at 3:1 proportion.

### 2.3. Larvae Kill Assay

The larval kill activity was followed by WHO [15] method. All doses ranged from 50 to 250*µ*g/ml and were tested on early third instars of the targeted mosquitoes. The plants products were solved in 1 ml dimethyl sulfoxide (DMSO) and diluted in 249 ml of dechlorinated water. Control was 1 ml of DMSO in 249 ml of dechlorinated water. Per each tested species, 25 individuals per replicate were stored in 249 ml of dechlorinated water and 1 ml of DMSO plus the required dose of mosquitocidal. Larval mortality was recorded after 24 h. For each dose, 5 replicates were carried out. Percent mortality was rectified for control mortality utilizing the formula by Abbott's [16].

### 2.4. Fourier Transform-Infrared Spectroscopy

Infrared analysis was used to probe bond vibrations and bending in molecules and to reveal the types of functional groups present in compound. Functional group region is in the range from 4000 to 1600 cm^−1^ and finger print region is from 2918 to 645 cm^−1^ [17].

### 2.5. Gas Chromatography-Mass Spectrometry Analysis

Gas chromatography-mass spectroscopic analysis of the leaf extracts was carried out on Agilent technologies (6890 N), JEOL GCMATE II, which comprised an autosampler and gas chromatography interfaced to a mass spectrometer (GC-MS) instrument employing the following condition: capillary column 624 ms (30m×0.32mm×1.8m) operating in an electron mode at 70 eV; helium (99.999%) was used as carrier gas at a constant flow of 1.491 mlmin^−1^ and injection volume of 1.0 ml, injector temperature of 140°C, and ion source temperature of 200°C. The oven temperature was programmed for 45°C. Mass spectra were taken at 70 eV [18].

### 2.6. Identification of Compound

Interpretation of mass spectrum of GC-MS was conducted using the databases of IIT, Chennai version (NIST08s) WILEY8, FAME. The spectrum of the unknown components was compared with the known components stored in the NIST08, WILEY8, FAME library. The name, molecular weight and structure of the components of the test materials were ascertained.

### 2.7. Statistical Analysis

Larvae mortality data were subjected to probit analysis [19] to calculate the LC_50_, LC_90_ utilizing statistical package of social science (SPSS) rendition 16.0 for Windows and data were analyzed using two-way ANOVA (factors: the tested extracts dose) followed by New Duncan test. The significance level was set at* P*<0.05.

## 3. Results

### 3.1. Larvae Kill Assay

Larvae kill assay of* E. variegata* was carried out with mosquito immature third instar larvae of* An. stephensi* and* Cx. quinquefasciatus*. The leaf extracts of* E. variegata* were 98.2% (methanol) at 250*µ*g/ml, and total percentage was 59.5%, 54.22% (ethyl acetate), 45.92% (chloroform), and 37.46% (hexane) larval mortality rate against* Cx. quinquefasciatus*. Moreover,* An. stephensi* was screened 92.8% (methanol) at 250*µ*g/ml, and total percentage was 55.02%, 47.74% (ethyl acetate), 37.94% (chloroform), and 28.74% (hexane) (Table 1). The larvae kill bioassay (LC_50_/LC_99_ values) of* E. variegata* extracts on* Cx. quinquefasciatus* was 137.67/297.33*µ*g/ml, sig. 0.001^b^, and R^2^ = 0.981; 148.65/316.67*µ*g/ml, sig. 0.005^b^, and R^2^ = 0.989; 178.12/380.83*µ*g/ml, sig. 0.081^b^, and R^2^ = 0.998; 206.31/418.47*µ*g/ml, sig. 0.290^b^, and R^2^ = 0.999 for methanol, ethyl acetate, chloroform, and hexane. In addition, the LC_50_/LC_99_ values were 157.69/339.55*µ*g/ml, sig. 0.126^b^, and R^2^ = 0.995; 180.07/377.66*µ*g/ml, sig. 0.533^b^, and R^2^ = 0.990; 222.21/456.18*µ*g/ml, sig. 0.376^b^, and R^2^ = 0.991; 234.01*µ*g/ml, sig. 0.386^b^, and R^2^ = 0.996, respectively, on* An. stephensi* (Table 2).

### 3.2. FT-IR Analysis of Methanol Extract

**Figure 2:**
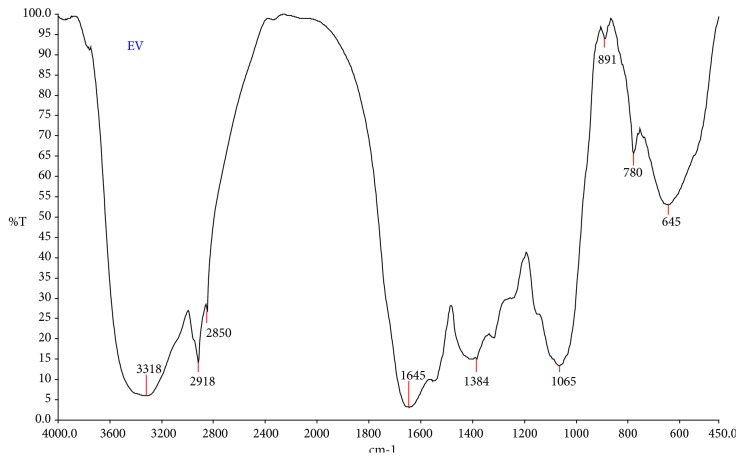
FT-IR spectrum of methanol leaf extract of* Erythrina variegata*.

FT-IR analysis was carried out to identify the functional groups of the methanol extract,* E. variegata*. FI-IR spectrum indicated the clear peaks with (3318, 2918, 2850, 1630, 1238, 1065, 891, 780, and 645 cm^−1^) different values (Figure 2). Above the peak values they corresponded to functional groups like, 1^*∗*^, 2^*∗*^ amines, amides group (N-H stretching 3318 cm^−1^), an alkenes group (C-H stretching 2918 and 2850 cm^−1^), a 1^*∗*^ amines group (N-H bend 1630 cm^−1^), aromatic amines group (C-N stretching 1238 cm^−1^), aliphatic amines group (C-N stretching 1065 cm^−1^), 1^*∗*^, 2^*∗*^ amines group (N-H wag 891 and 780 cm^−1^), and alkyl halides group (C-Br stretching 654 cm^−1^). The functional groups such as alcohol, amines, amides, alkenes, 1^*∗*^ amines, aromatic amines, aliphatic amines, 1^*∗*^,2^*∗*^ amines, and alkyl halides confirmed their presence in methanol extract.

### 3.3. GC-MS Analysis of Methanol Extract

**Figure 3:**
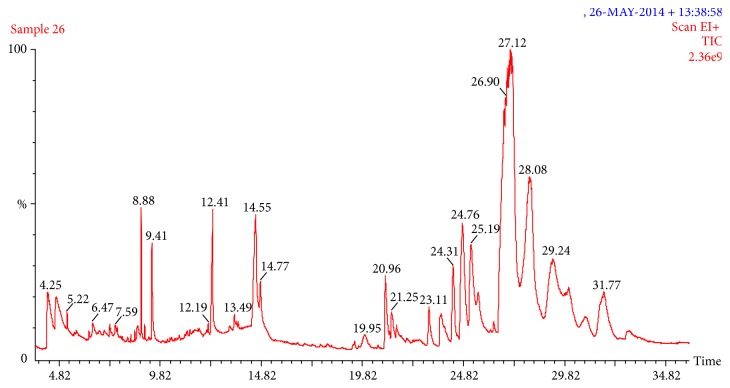
GC-MS chromatogram of* Erythrina variegata* methanol leaf extract.

Twenty-five compounds and the total concentration of percentage 106.95% from leaf methanol extract were noticed (Table 3 and Figure 3), particularly some amount of compounds, 12-Octadecenoic acid, methyl ester (36.32%), Prednisone (11.88%), Cymarin (8.40%), 9,12-Octadecadienoic acid (Z,Z) (5.63%), 4-(3,4-Dimethoxyphenyl)-2-methoxy-6-phenylpyrimidine (5.13%), 6-Azabicyclo[3.2.1]octane-1,2,2-tricarbonitrile, 5-amino-3-(4-methoxyphenyl)-7-oxo-4-propyl- (4.93%), 2-Furancarboxaldehyde, 5-(hydroxymethyl) (4.08%), 2H-1-Benzopyran-2-one, 4-hydroxy-3-(1,2,3,4-tetrahydro-1-naphthalenyl)- (3.99%), 4-Piperidinamine, N,1-dimethyl- (3.38%), n-Hexadecanoic acid (2.35%), 2,6-Bis(4-methoxybenzylidene) cyclohexanone (2.16%), 6-Benzylamino-1-methyl-5-nitro-2-phenyl-1H-pyrimidin-4-thion (1.90%), 1-(3a-Hydroxy-1-methyl-2-thioxo-2,3,3a,8a-tetrahydro-1H-1,3,8-triaza-cyclopenta[a]inden-8-yl)-ethanone (1.89%), 2H-1-Benzopyran, 6,7-dimethoxy-2,2-dimethyl- (1.37%), N-[2-Hydrazono-4-(4-methoxyphenyl)-1H,2H-pyrimidin-1-yl]-benzamide (1.05%), and Ascaridole epoxide (1.01%).

### 3.4. Mass Spectral Analysis of Methanol Extract

**Figure 4:**
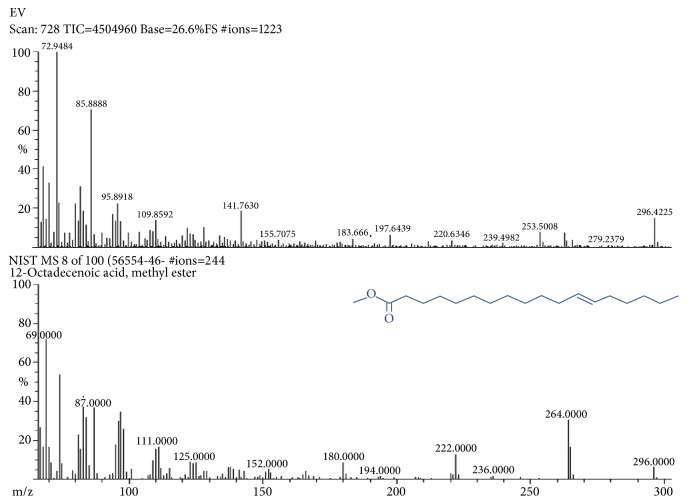
Mass spectra of 12-Octadecenoic acid, methyl ester compound in the* Erythrina variegata* methanolic leaf extract.

**Figure 5:**
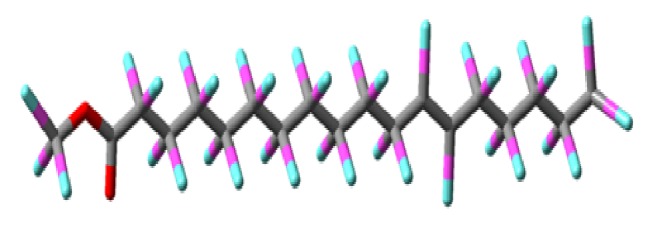
3D 12-Octadecenoic acid, methyl ester compound structure of* Erythrina variegata* leaf methanol extract.

Mass spectral analysis was observed to be m/z value 296.487 for 12-Octadecenoic acid, methyl ester compound consistent with the proposed molecular formula (C_19_H_36_O_2_) of the compound (Figure 4). Mass spectral studies strongly confirmed that 12-Octadecenoic acid, methyl ester structure was present in the methanol extract. The 12-Octadecenoic acid, methyl ester* 3D* structure contained some molecules (Figure 5). Molecules consisting of carbon 19 atoms (gray), hydrogen 36 atoms (greenish blue), and oxygen 2 atoms (red) were indicated by different colors.

## 4. Discussion

In present day, mosquito life cycle control using larval and eggs activity agents is a major compound in the control of vector borne diseases. Medicinal plant as potential larval and eggs activity is considered as viable and preferred alternative in the life cycle control of the mosquitoes at the community level. Phytochemicals derived from plants act as general toxicants against adults as well as against larval stages of mosquito vectors, while some act as growth inhibitors or as chemosterilant or act as repellant or attractants. In the background of resistance developed by the mosquitoes against chemical pesticides, identifying new larvicidal compounds from natural plants products against mosquitoes is noticed [20, 21]. Biological active compounds have been utilized to the improvement of eco-friendly vector management and botanical pesticides are cheap and easy to administer against mosquito management [22, 23]. A large number of plant extracts have been reported to have mosquitocides or repellent activities against mosquito vectors, but very few plant products have shown practical utility for mosquito control [24]. Therefore, the present study is focused on the larvicidal activity of* E. variegata* leaf extract tested against* An. stephensi* and* Cx. quinquefasciatus*. Present results showed that the larvicidal activity (LC_50_ and LC_90_) of* E. variegata* extracts on* Cx. quinquefasciatus* was found to be 137.67/297.33*µ*g/ml, 148.65/316.67*µ*g/ml, 178.12/380.83*µ*g/ml, and 206.31/418.47*µ*g/ml for methanol, ethyl acetate, chloroform, and hexane, respectively. The present researches are comparable with earlier reports [25, 26]. As an evidence, the LC_50_ and LC_90_ of citronella component from* Melissa officinalis* tested against* An. stephensi* were 85.44 and 159.73 mg/L [27]. Furthermore, the larvicidal activity of* Gymnema sylvestre* tested against* Cx. tritaeniorhynchus* with LC_50_ values of acetone, chloroform, and methanol extracts were 34.756, 31.351, and 28.577 mg/ml, respectively [28].

Insecticidal activity of piperitenone oxide compound from* Mentha* plants have been studied by Mohamed and Abdegaleil [29] and reported significantly effect on stored grain pests,* Sitophilus oryzae* and* Tribolium castaneum*. Further, mosquitocidal activity of piperitenone oxide compound tested against* An. stephensi* and* Cx. quinquefasciatus* have been reported by many authors [30, 31]. Major phytochemical compound, namely, phytol isomer in chloroform extract of* Terminalia chebula* leaves, has potential mosquito larvicides and repellents on* Cx. quinquefasciatus* [32]. Different compounds of* Spathodea campanulata* by GC-MS analysis reported that a major component is phytol isomer identified as mosquitocidal activity against* Ae. aegypti* [33]. This plant possessed some phytochemical properties such as spathodol, caffeic acid, other phenolic acids, and flavonoids [34]. These chemicals have been affecting the mitochondria [35], midgut epithelium, gastric caeca and malpighian tubules of mosquito larvae [36, 37]. Similarly, the bioactive compounds from peel extracts of* Arachis hypogaea* showed higher efficiency in reducing mosquito menace due to their larvicidal toxicity [38]. The maximum amount of flavonoid (44.6%) was noticed in acetone extract of* Andrographis paniculata* followed by phenol (32.2%), alkaloid (22.2%), steroid (20.5%), chlorogenic acid (5.3%), and tannin (3.7%) [39]. Plant-borne compounds and the fractions were tested as larvicides, ovicides, and repellency against* An. stephensi*,* Ae. Aegypti,* and* Cx. quinquefasciatus*. The larvicides activity was tested by 11-octadecenoic acid, methyl ester compound against* Cx. quinquefasciatus*,* An. Stephensi,* and* Ae. aegypti* with LC_50_ values of 20.51, 22.32, and 23.90 ppm [40]. The highest larvicidal activity of important medicinal plants,* Sesamum indicum*,* Pongamia pinnata,* and* Croton bonplandianum *methanol extract with LC_50_ and LC_95_ values were 108.55 and 230.57 ppm, 143.59 and 305.52 ppm, and 154.51 and 319.01 ppm, respectively [41].

## 5. Conclusion

One isolated compound and 12-Octadecenoic acid, methyl ester were more active than the methanol extract on* Cx. quinquefasciatus*. A possible explanation could be that the interaction of the compounds with other constituents of the methanol extract could be responsible for the higher mosquito larval killing activity observed in the methanol extract as against the* Cx. quinquefasciatus* exhibited by the identification of compounds. Further research on the isolation and purification of active compounds from the leaves should be conducted to increase its therapeutic applications.

## Figures and Tables

**Table 1:** Percentage mortality of mosquito larvae of *An. stephensi *and* Cx. quinquefasciatus* exposed to different concentrations of different solvent leaf extracts of *E. variegata*.

Solvents	Mortality rate (%) (Mean ± SD^a^)		
	Concentration (ppm)	*An. Stephensi*	*Cx. quinquefasciatus*
Hexane	Control	0^a^	0^a^
	50 *μ*g/ml	3.20 ±1.7 (10.2%)^a^	6.60 ±1.8 (13.3%)^a^
	100 *μ*g/ml	8.80 ±2.6 (17.8%)^a^	11.80±2.2 (23.9%)^a^
	150 *μ*g/ml	16.20±2.7 (24.7%)^a^	23.60±1.7 (38.4%)^ab^
	200 *μ*g/ml	34.60±2.6 (36.8%)^b^	42.80±2.2 (48.2%)^bc^
	250 *μ*g/ml	62.80±2.7 (54.2%)^cd^	73.40±1.7 (63.5%)^cd^
Total percentage of death rate (%)		28.74%	37.46%
Ethyl acetate	Control	0^a^	0^a^
	50 *μ*g/ml	8.80±2.4 (24.2)^a^	14.40±3.8 (24.2)^a^
	100 *μ*g/ml	15.60±3.1 (35.6)^b^	22.80±2.1 (37.9)^ab^
	150 *μ*g/ml	31.80±2.3 (46.9)^c^	40.80±3.6 (48.7)^bc^
	200 *μ*g/ml	60.20±2.3 (62.7)^cd^	74.20±3.5 (63.5)^cd^
	250 *μ*g/ml	81.40±1.7 (69.3)^d^	97.60±0.8 (96.8)^d^
Total percentage of death rate (%)		47.74%	54.22%
Chloroform	Control	0^a^	0^a^
	50 *μ*g/ml	5.40 ±2.6 (15.2)^a^	9.80±2.6 (20.3)^a^
	100 *μ*g/ml	12.80±2.4 (24.9)^a^	19.20±3.2 (31.8)^a^
	150 *μ*g/ml	20.60±2.5 (41.5)^b^	32.60±2.2 (44.5)^ab^
	200 *μ*g/ml	36.20±2.5 (51.7)^c^	53.20±2.5 (58.4)^bc^
	250 *μ*g/ml	65.80±2.1 (56.4)^d^	86.20±2.1 (74.6)^cd^
Total percentage of death rate (%)		37.94%	45.92%
Methanol	Control	0^a^	0^a^
	50 *μ*g/ml	12.20±3.2 (26.2)^a^	16.80±2.7 (29.3)^a^
	100 *μ*g/ml	21.80±2.5 (36.8)^b^	26.20±2.6 (42.5)^ab^
	150 *μ*g/ml	39.80±2.6 (51.6)^c^	45.60±3.8 (54.8)^bc^
	200 *μ*g/ml	68.20±3.9 (67.7)^cd^	81.40±1.9 (72.7)^cd^
	250 *μ*g/ml	92.60±1.6 (92.8)^d^	100.0±0.0 (98.2)^d^
Total percentage of death rate (%)		55.02%	59.5%

^a^Values are mean ± SD of four replicates. Within each row, different letters indicate significant differences (ANOVA, New Duncan test, and P<0.05).

**Table 2:** Larvicidal activity of *E. variegata *extracts against *An. stephensi *and *Cx. quinquefasciatus*.

Species	Solvents	LC_50_ (*μ*g/ml) 24 h	LC_99_ (*μ*g/ml) 24 h	*χ* ^2^ values (df^a^)	*R* ^2^ values	Significant
*An. stephensi*	Hexane	234.01^d^	449.51^d^	3.03 (3)^a^	y= 4.014 +9.585^*χ*^	0.386^b^
					(0.996)	
	Ethyl acetate	180.07^b^	377.66^b^	2.19 (3)^a^	y= 2.928 +1.408^*χ*^	0.533^b^
					(0.990)	
	Chloroform	222.21^c^	456.18^c^	3.10 (3)^a^	y= 3.728 +7.951^*χ*^	0.376^b^
					(0.991)	
	Methanol	157.69^a^	339.55^a^	5.72 (3)^a^	y= 2.857 +3.577^*χ*^	0.126^b^
					(0.995)	
*Cx. Quinquefaciatus*	Hexane	206.31^d^	418.47^d^	3.74 (3)^a^	y= 4.157 +8.482^*χ*^	0.290^b^
					(0.999)	
	Ethyl acetate	148.65^b^	316.67^b^	13.00 (3)^a^	y= 3.242 +2.322^*χ*^	0.005^b^
					(0.989)	
	Chloroform	178.12^c^	380.83^c^	6.73 (3)^a^	y= 3.885 +4.634^*χ*^	0.081^b^
					(0.998)	
	Methanol	137.67^a^	297.33^a^	16.21 (3)^a^	y= 2.485 +7.245^*χ*^	0.001^b^
					(0.981)	

Values represent mean of five replications; mortality of the after 24 h of exposure period; LC_50_= lethal concentration brings out 50% mortality; LC_99_= lethal concentration brings out 99% mortality; different letters (a, b, c, and d) within the same row; *R*^*2*^ = regression; **χ**^2^ = chi-squire; df^a^ = degree of freedom; ^b^significant at *p*<0.05.

**Table 3:** Phytochemical components identified in the leaf powder sample (Code No. 26) (GC MS study).

MF	Name of the compound	RT	MW	Content %	MI
C_7_H_16_N_2_	4-Piperidinamine, N,1-dimethyl-	4.25	128	3.38	*RI, MS*
C_6_H_6_O_3_	2-Furancarboxaldehypde, 5-(hydroxymethyl)-	4.68	126	4.08	*RI, MS*
C_10_H_16_O_3_	Ascaridole epoxide	5.22	184	1.01	*RI, MS*
C_11_H_22_O_2_	2-Pentyl-cyclohexane-1,4-diol	6.47	186	0.70	*RI, MS*
C_10_H_13_NO_3_	L-Serine, O-(phenylmethyl)-	7.32	195	0.41	*RI, MS*
C_15_H_26_O	tau.-Cadinol	8.88	222	0.76	*RI, MS*
C_15_H_26_O	Epiglobulol	9.05	222	0.09	*RI, MS*
C_13_H_16_O_3_	2H-1-Benzopyran, 6,7-dimethoxy-2,2-dimethyl-	9.41	220	1.37	*RI, MS*
C_10_H_13_NO_3_	L-Serine, O-(phenylmethyl)-	12.19	195	0.12	*RI, MS*
C_16_H_32_O_2_	n-Hexadecanoic acid	12.41	256	2.35	*RI, MS*
C_12_H_22_O_11_	à-D-Glucopyranose, 4-O-á-D-galactopyranosyl-	13.49	342	0.30	*RI, MS*
C_18_H_32_O_2_	9,12-Octadecadienoic acid (Z,Z)-	14.55	280	5.63	*RI, MS*
C_15_H_18_O_3_	à-Santonin	19.95	246	0.97	*RI, MS*
C_12_H_13_N_3_O_2_S	1-(3a-Hydroxy-1-methyl-2-thioxo-2,3,3a,8a-tetrahydro-1H-1,3,8-triaza-cyclopenta[a]inden-8-yl)-ethanone	20.96	263	1.89	*RI, MS*
C_21_H_34_O_3_S_2_	18-Hydroxy-10-pentyl-11-oxa-1,5-dithia-spiro[5.13]nonadec-15-yn-12-one	21.25	398	0.76	*RI, MS*
C_18_H_17_N_3_O_2_	Isoindole-1,3(1H,3H)-dione, 2-[2-(4-methylphenylhydrazono)propyl]-	21.51	307	0.43	*RI, MS*
C_18_H_17_N_5_O_2_	N-[2-Hydrazono-4-(4-methoxyphenyl)-1H,2H-pyrimidin-1-yl]-benzamide	23.11	335	1.05	*RI, MS*
C_18_H_16_N_4_O_2_S	6-Benzylamino-1-methyl-5-nitro-2-phenyl-1H-pyrimidin-4-thion	23.70	352	1.90	*RI, MS*
C_22_H_22_O_3_	2,6-Bis(4-methoxybenzylidene)cyclohexanone	24.31	334	2.16	*RI, MS*
C_19_H_18_N_2_O_3_	4-(3,4-Dimethoxyphenyl)-2-methoxy-6-phenylpyrimidine	24.76	322	5.13	*RI, MS*
C_19_H_16_O_3_	2H-1-Benzopyran-2-one, 4-hydroxy-3-(1,2,3,4-tetrahydro-1-naphthalenyl)-	25.19	292	3.99	*RI, MS*
C_19_H_36_O_2_	12-Octadecenoic acid, methyl ester	27.12	326	36.32	*RI, MS*
C_21_H_26_O_5_	Prednisone	28.08	358	11.88	*RI, MS*
C_30_H_44_O_9_	Cymarin	29.24	548	8.40	*RI, MS*
C_20_H_21_N_5_O_2_	6-Azabicyclo[3.2.1]octane-1,2,2-tricarbonitrile, 5-amino-3-(4-methoxyphenyl)-7-oxo-4-propyl-	31.77	363	4.93	*RI, MS*

MF, molecular formula; RT, retention time; MW, molecular weight; MI, mode of identification.

## Data Availability

The data used to support the findings of this study are available from the corresponding author upon request.
